# Tumour suppressor gene (CDKNA2) status on chromosome 9p in resected renal tissue improves prognosis of localised kidney cancer

**DOI:** 10.18632/oncotarget.12196

**Published:** 2016-09-22

**Authors:** Ismail El-Mokadem, Thomas Kidd, Norman Pratt, Stewart Fleming, Ghulam Nabi

**Affiliations:** ^1^ Academic Section of Urology, Division of Cancer Research, University of Dundee, Ninewells Hospital, DD1 9SY, Dundee, Scotland; ^2^ Department of Cytogenetic, University of Dundee, Ninewells Hospital, DD1 9SY, Dundee, Scotland; ^3^ Department of Pathology, University of Dundee, Ninewells Hospital, DD1 9SY, Dundee, Scotland

**Keywords:** kidney cancer, genetics, microsatellite analysis, chromosome 9p

## Abstract

**Background:**

Genetic alterations on chromosome 9p, including inactivation of the tumour suppressor gene, CDKN2A, result in cellular proliferation and growth of tumours. Our aim was to use microsatellite analysis and fluorescence *in situ* hybridization (FISH) to characterise the architecture of this region.

**Results:**

Seventy-five out of 77 clear cell renal cell cancers (tumour/normal pairs) were interpretable for LOH analysis on chromosome 9p (two tumours were excluded, as all five primers were uninformative). Twenty out of 75 (26.6%) tumours showed LOH in at least one of the five primers employed. Most allelic deletions were detected, telomeric to the CDKN2A region at D9S916, with 11 out of 52 informative tumours (21%) displaying LOH. The LOH in the coding region of CDKN2A, at D9S974 and D9S942, was associated with a higher pT-stage (*p* = 0.004) and metastasis (*p* = 0.006, both markers). The rate of chromosome 9p deletion in ccRCC was 44% (35/80 cases) according to FISH. Somatic copy number loss of chromosome 9p was associated with a larger tumour size (*p* = 0.002), higher pathological tumour stage (*p* = 0.021), presence of tumour necrosis (*p* = 0.019) and microvascular invasion (*p* = 0.032). The cases with copy number loss, loss of heterozygosity and copy number neutral (*n* = 42) were at a higher risk of cancer-specific death when compared to tumours in category D (*n* = 32) (Log-rank: *p* = 0.001). Seventeen patients with localised ccRCC developed recurrence, and fourteen of those showed either LOH or somatic copy number loss at CDKN2A (Log-rank: *p* = 0.005). Multivariate analysis showed that LOH or copy number loss at CDKN2A retained its independent prognostic effect, improving the predictive accuracy of stage and SSIGN score by concordance Index C from 0.823 to 0.878 (*p* = 0.001).

**Materials and Methods:**

Cytogenetics data, microsatellite analysis and FISH were acquired for a cohort of patients undergoing resection for clinically localised renal cancer between January 2001 and December 2005. Five microsatellite markers (D9S916, D9S1814, D9S974, D9S942 and D9S171) assessed loss of heterogeneity (LOH) using DNA samples and in the same cohort FISH analysis was accomplished on tissue microarray slides. The FISH data were scored by two observers blinded to the histological data of the patients. Cytogenetic aberrations were correlated with histological and clinical outcomes by univariate and multivariate analyses using different prognostic models. Disease specific and recurrence free survival based on cytogenetic changes were assessed by Kaplan Meier methods.

**Conclusions:**

A comprehensive cytogenetic analysis using microsatellite analysis and FISH of the CDKN2A region on chromosome 9p improves the predictive accuracy of known prognostic factors in clinically localised renal cell carcinoma undergoing surgical resection.

## INTRODUCTION

Loss of heterozygosity (LOH) and copy number deletion are two different cytogenetic aberrations which can result in down-regulation or inactivation of a gene. These two mechanisms could be involved in tumour progression if they result in inactivation of tumour suppressor genes, such as CDKN2A/B located on chromosome 9p.

CDKNA region contains genetic information for the synthesis of many tumour suppressor proteins such as p16 ^INK4a^, p14^ARF^, and p15^INK4b.^ These proteins regulate progression of cellular proliferation through cell cycle, for example p16INK4a plays a key role in transition of cell from G1-S phase in the cell cycle through p16-Rb pathway. In addition, this plays an active role in cellular senescence, p16 through interconnections influences tumor suppressor P53 pathways [1, 2] Any alteration in this region (mutation or hypermethylation) can potentially lead to reduced or no synthesis of these proteins and hence unregulated growth of cells [3].

Several cytogenetic methods, such as fluorescence *in situ* hybridization (FISH), comparative genomic hybridization (CGH) and microsatellite analysis, have been reported; each has its advantages and disadvantages. A recent review of published literature on prognostic value of 9p deletion in RCC has shown that most studies were observational and used a wide variety of molecular and conventional cytogenetic techniques. The studies were mostly old series with no common protocols, which led to a discrepancy in the outcomes and the quality of the studies reviewed [4].

Three studies have shown that somatic copy number loss involving 9p can be associated independently with worse outcomes in ccRCC. All three studies relied on I-FISH to assess loss of chromosome 9p; however, the criteria and cut-off values used to define deletion differed among the studies, as did the probes employed [5–7]. Our group used I-FISH to carry out a validation study and confirmed the above findings in patients with surgically resected RCC that had the longest follow-up [8]. The probe employed was a dual-colour probe with a locus-specific identifier on the CDKN2A gene.

By contrast, studies using microsatellite analysis have demonstrated that LOH on chromosome 9p is associated with adverse histological features and a risk of progression of renal cancer [9–11]. Microsatellite analysis, using paired control normal renal tissue and tumour DNA, is a sensitive technique for detecting LOH in tumours. It is a fast and inexpensive analytical method and can be performed on degraded DNA extracted from formalin-fixed, paraffin-embedded tissue. Kinoshita et al. were the first to propose that LOH in the region of 9p21 was associated with progression of RCC and metastasis [9], but the study lacked long-term follow-up. Only a few studies correlated the allelic deletion of 9p and survival with varied follow-up duration which ranged between 31 and 48 months [11–13]. Some studies relied on only one or two microsatellites telomeric to 9p21 for assessment of allelic deletion of 9p and could therefore have missed more centromeric LOH involving regions harbouring several tumour suppressor genes involved in cell cycle regulation.

In comparison to I-FISH, microsatellite karyotyping detects LOH in the absence of copy number variation (deletion). This phenomenon is also known as copy number neutral LOH (CNNLOH) and was previously called uniparental disomy (UPD). Studies on oesophageal cancer have shown that copy number neutral LOH is a common phenomenon [14, 15] The literature contains few reports on CNN-LOH in clear cell RCC especially in the region of chromosome 9p21, which harbours CDKN2A/B, one of the main tumour suppressor genes. CNN-LOH could therefore be an underestimated event which could result in gene inactivation due to duplication of the mutated or non-functioning allele.

The objective of the present study were:

- to assess the concordance between microsatellites and I-FISH, including the diagnostic utility of the individual methods.
- to determine whether the overall prognostic impact of a 9p deletion could be improved using a strategy incorporating FISH and LOH analysis, particularly in conjunction with long-term follow-up.

## RESULTS

### Cohort characteristics

The study population included 89 (82.4%) patients with clear cell histology, 13 (12%) with papillary renal cell carcinoma (pRCC), 5 (4.6%) with chromophobe appearance and one (1%) with collecting duct carcinoma. The clear cell subtype was the focus of this study; therefore, somatic copy number loss and LOH in the 9p region were analysed in relation to adverse histopathological factors and survival only in this subtype. However, we included all the subtypes in the concordance analysis of LOH between microsatellites and I-FISH. The mean age of patients with ccRCC was 62.8 (range 38–84) with a mean follow-up of 80 months (range 6–165 months).

### Microsatellite analysis of LOH at the 9p21 region and correlation with pathological parameters in ccRCC

In total, 96 out of 108 cases represented on the tissue microarrays (TMAs) were tested for LOH with 5 markers. Four markers (D9S916, D9S974, D9S942 and D9S1814) were within the 9p21 region and 1 marker (D9S171) was within 9p13 region. Seventy-seven (80%) tumours were clear cell subtype, 13 (13.5%) were papillary RCC, 5 (5.2%) were chromophobe RCC, and only one was collecting duct RCC.

Except for two uninformative cases, 75 out of 77 ccRCC tumour/normal pairs were interpretable for LOH analysis on chromosome 9p (Figure 1). Average tumour size for these patient was 6.3 cm. The mean age in this cohort was 62.5 years old. The median follow-up was 91.8 months (mean 85 months). Again, details of allelic deletions have been reported previously [16].

**Figure:**
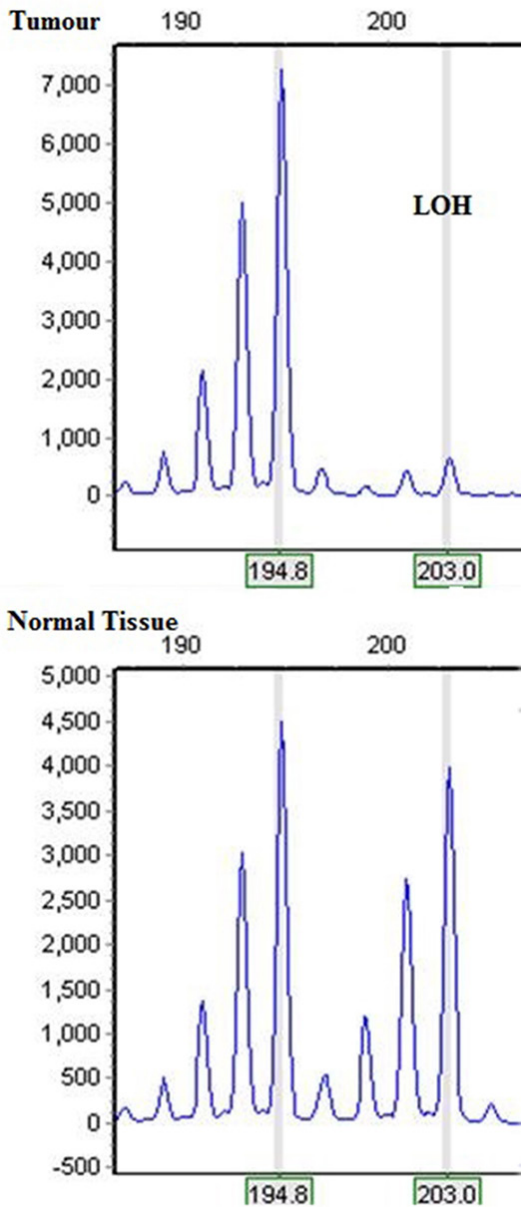
Microsatellite analysis is showing allelic deletion in tumour 81 involving more than one marker

In addition to previous publication, LOH at D9S916 was statistically associated with a higher pT stage (*p* = 0.005), sarcomatoid changes (*p* = 0.026), and renal sinus invasion (*p* = 0.014), with only an observed trend for metastasis (*p* = 0.10).

The LOH within the coding region of CDKN2A, at D9S974 and D9S942, was also associated with higher pT stage (*p* = 0.004 and *p* = 0.003, respectively and metastasis (*p* = 0.006, both markers). The LOH within D9S974 and D9S942 was also associated with renal sinus invasion (*p* = 0.015 and *p* = 0.006, respectively.

Almost half the cases were uninformative at D9S1814, but the LOH at this locus was associated only with higher pT stage (*p* = 0.028). A trend was also observed with renal sinus invasion (*p* = 0.059).

On the other hand, LOH was the only marker within 9p13 (D9S171) and showed a correlation with renal sinus invasion only (*p* = 0.027). A trend was also noted with pathological tumour stage (*p* = 0.083).

### Fluorescence *in-situ* hybridisation and correlation with pathological parameters

Of the 108 tumours, 98 had valid 9p scoring on the TMA. Eighty of these 98 cases were ccRCC subtype and were included in the survival analysis in this study. Twelve were papillary RCC, five were chromophobe RCC and only one was a collecting duct carcinoma.

A complete description of the results of this experiment was previously reported [8, 7]. Based on I-FISH interpretation using pre-set criteria, the rate of chromosome 9p deletion in ccRCC was 44% (35/80 cases). Somatic copy number loss of chromosome 9p was associated with a larger tumour size (*p* = 0.002), a higher pathological tumour stage (*p* = 0.021), the presence of tumour necrosis (*p* = 0.019) and microvascular invasion (*p* = 0.032).

### Concordance between LOH on microsatellite analysis and fluorescence *in-situ* hybridization in all RCC specimens

Ninety-six cases (paired tumour and normal tissue) were assessed for microsatellite analysis and included in the concordance analysis for 9p loss using FISH.

Of the 68 cases with informative markers within the coding region of CDKN2A (D9S974 and D9S942), LOH was observed in 17 tumours (25.7%), with 7 tumours showing LOH at both microsatellites and 10 tumours showing LOH for one of the two markers.

Sixty-three of the 68 informative cases had valid I-FISH scores available for comparison. Four tumours were excluded from concordance analysis as they were marked as a homozygous loss by I-FISH. In total, 59 tumours were available for concordance analysis between microsatellites and I-FISH. Both techniques agreed on 30 tumours showing no LOH or copy number loss at CDKN2A. LOH within CDKN2A region was detected and agreed upon by both the techniques in 6 tumours. Overall, the concordance rate between microsatellites and I-FISH was 61% (36/59).

On the other hand, microsatellite analysis showed LOH at CDKN2A in 9 tumours that did not show copy number loss on FISH. This finding could be explained by the common phenomenon of copy number neutral LOH (CNNLOH). The rate of CNNLOH at the CDKN2A coding region was 60% (9/15).

A further analysis of 14 tumours showing 9p loss using I-FISH, with an average pooled percentage of abnormal nuclei of 49% (range: 39–60%), did not show allelic loss on microsatellite analysis. As a result, the sensitivity of microsatellite analysis to detect copy number loss LOH was poor (30%; 6/20) when compared to FISH.

### Combined copy number loss and loss of heterozygosity on chromosome 9p and its impact on prognosis in ccRCC

The data of copy number loss using FISH and from microsatellite analysis on the LOH in the CDKN2A region were combined to assess the impact of aberrations on the outcomes of ccRCC. Seventy-four tumours with complete information were divided into four categories:

- Tumours with somatic copy number loss (*n* = 29).
- Tumours with somatic copy number loss and LOH (*n* = 6).
- Tumours with copy number neutral LOH (*n* = 7).
- Tumours with normal copy number and heterozygous (*n* = 32).

**Figure 2:**
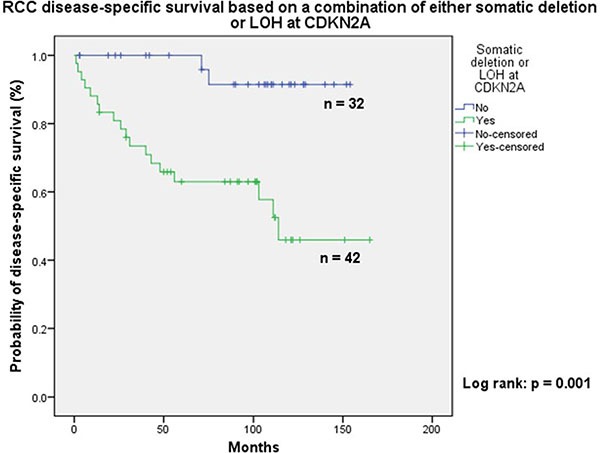
Kaplan Meir analysis clearly showing poor disease specific survival in patients with cytogenetic abnormalities on chromosome 9p

The cases in the first three groups combined (*n* = 42) were at a higher risk of cancer-specific death when compared to tumours in category D (*n* = 32) (Figure 2: Log-rank: *p* = 0.001). Seventeen patients with localised ccRCC developed recurrence; fourteen of these showed either LOH or somatic copy number loss at CDKN2A (Figure 3: Log-rank: *p* = 0.005).

**Figure:**
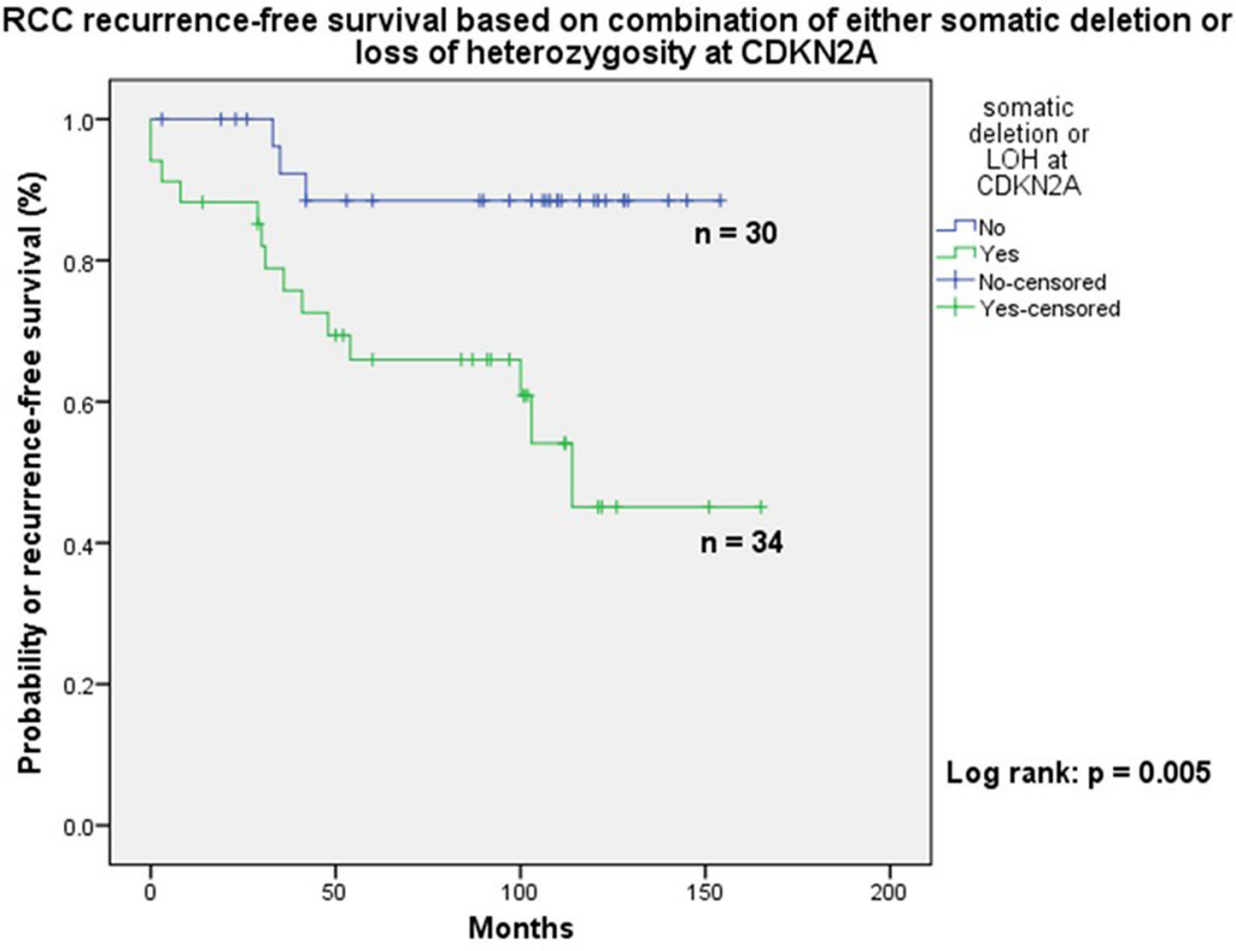
Showing better recurrence free survival in patients with no loss of heterozygosity or deletion of chromosome 9p

Univariate Cox-proportional hazard analysis revealed that cases with LOH or copy number loss at CDKN2A showed a higher hazard ratio when compared to each aberration independently for disease-specific survival (DSS) and recurrence-free survival (RFS) (Table 1). All cases were categorised based on LOH status and copy number loss at CDKN2A in relation to validated prognostic nomograms for metastasis or RFS and DSS in ccRCC (Table 2).

**Table 1:** Concordance between FISH and microsatellite analyses in the region of CDKN2A

Concordance Analysis		I-FISH		
		No Deletion	Monosomy/LOH	Total
LOH analysis with microsatellites	No	30	14	44
	Yes	9	6	15
	Total	39	20	59

**Table 2:** Univariate Cox proportional hazard model

Univariate analysis for disease-specific survival (DSS)					
Variable	Categories	*p*-value	Hazard Ratio	95% CI	
				Lower	Upper
**LOH at CDKN2A**	Yes vs. No	***0.005***	5.374	1.644	17.564
**Somatic deletion at CDKN2A**	Yes vs. No	***0.002***	5.703	1.904	17.077
**Combined deletion or LOH at CDKN2A**	Deletion or LOH vs. normal	***0.004***	***8.464***	1.958	36.578
**Univariate analysis for recurrence-free survival (RFS)**					
**Variable**	**Categories**	***p*-value**	**Hazard Ratio**	**95% CI**	
				**Lower**	**Upper**
**LOH at CDKN2A**	Yes vs. No	***0.023***	4.040	1.213	13.460
**Somatic deletion at CDKN2A**	Yes vs. No	***0.003***	5.365	1.763	16.327
**Combined deletion or LOH at CDKN2A**	Deletion or LOH vs. normal	***0.007***	***5.620***	1.670	32.674

Multivariate analysis for DSS revealed that LOH or copy number loss at CDKN2A retained its independent prognostic effect with pathological T stage and state of metastasis in model 1 (Table 3). It also remained as an independent prognostic factor with the Mayo Clinical Stage, Size, Grade and Necrosis (SSIGN) score in model 2, improving its predictive accuracy expressed by concordance Index C from 0.823 to 0.878 (*p* = 0.001).

**Table 3:** Association between copy number loss and LOH on CDKN2A with SSIGN and Leibovich scores

		Copy number loss at CDKN2A		LOH at CDKN2A		LOH or copy number loss at CDKN2A	
		Deletion	No deletion	LOH	Normal	Yes	No
**SSIGN score**	**Low (0–3)**	10	25	5	28	13	19
	**Intermediate (4–7)**	15	13	5	18	17	8
	**High (8–13)**	10	7	3	8	12	5
	**Total**	**35**	**45**	**13**	**54**	**42**	**32**
**Leibovich score**	**Low (0–2)**	5	20	2	21	7	16
	**Intermediate (3–5)**	12	13	6	17	15	8
	**High (6–11)**	12	8	2	13	12	6
	**Total**	**29**	**41**	**10**	**51**	**34**	**30**

On the other hand, multivariate analysis for RFS showed that combination of copy number loss or LOH at CDKN2A is an independent prognostic factor for recurrence in ccRCC in addition to the pT stage in model 3 (Table 4). The integration of copy number loss or LOH at CDKN2A with the Leibovich score enhanced its predictive accuracy expressed by concordance Index C from 0.734 to 0.801 (*p* = 0.009).

**Table 4:** Multivariate Cox proportional hazard model analysis for RFS and DSS

Model 1 for RCC disease-specific survival (DSS)					
Variable	Categories	*p*-value	Hazard Ratio	95% CI	
				Lower	Upper
**Combined deletion or LOH at CDKN2A**	Deletion or LOH vs. normal	***0.045***	4.568	1.031	20.238
**T-stage**	pT3/4 vs. pT1/2	***0.006***	6.724	1.710	26.435
**Metastasis**	N+M+ vs. N0M0	***0.003***	4.532	1.678	12.241
**Model 2 for RCC disease-specific survival (DSS)**					
**Variable**	**Categories**	***p*-value**	**Hazard Ratio**	**95% CI**	
				**Lower**	**Upper**
**Combined deletion or LOH at CDKN2A**	Deletion or LOH vs. normal	***0.010***	6.808	1.567	29.567
**SSIGN score**	3 sub-categories	***< 0.001***	4.361	2.163	8.974
**Model 3 for recurrence-free survival (RFS)**					
**Variable**	**Categories**	***p*-value**	**Hazard Ratio**	**95% CI**	
				**Lower**	**Upper**
**T-stage**	pT3/4 vs. pT1/2	***0.007***	4.188	1.491	11.761
**Combined deletion or LOH at CDKN2A**	Deletion or LOH vs. normal	***0.023***	5.701	1.265	25.701
**Model 4 for recurrence-free survival (RFS)**					
**Variable**	**Categories**	***p*-value**	**Hazard Ratio**	**95% CI**	
				**Lower**	**Upper**
**Combined deletion or LOH at CDKN2A**	Deletion or LOH vs. normal	***0.019***	5.968	1.347	26.435
**Leibovich score**	3 sub-categories	***0.006***	3.061	1.387	6.758

## DISCUSSION

This study has confirmed previous findings where the LOH on chromosome 9p in clear cell RCC is associated with adverse histopathological features and worse prognosis, but it also showed enhanced prognostic significance when LOH is combined with FISH analysis.

The detection rate of allelic deletion-as previously reported- on chromosome 9p in ccRCC by [16] studies using microsatellites [10, 12, 13] and more contemporary studies relying on SNP arrays [21, 22].

The LOH within the coding region of CDKN2A was associated with higher tumour grade and a higher risk of metastasis. The frequency of LOH for all 4 markers on 9p21 (D9S916, D9S974, D9S942 and D9S1814) was significantly associated with locally advanced tumours, highlighting our previous observation that LOH could be a biomarker for aggressive renal cell cancer. The LOH on 9p21, especially the markers within the region of CDKN2A in addition to D9S916, were all independent prognostic factors in multivariate analysis models when integrated separately for DSS. In the group of clinically localised ccRCC, the LOH in the CDKN2A region was associated with a higher risk of recurrence or development of metastatic disease, as determined by univariate analysis (*p* = 0.023; HR 4.04). This LOH remained an independent prognostic factor in the multivariate analysis model including the Leibovich score (*p* = 0.014; HR = 4.7).

These findings confirm again that LOH involving chromosome 9p21, and particularly LOH at the coding region of CDKN2A, portends a worse prognosis in ccRCC in long term follow up and they validated the findings from I-FISH-based analysis of 9p deletion.

Previous studies have suggested that the LOH on chromosome 9p is associated with adverse histological features and risk of progression of renal cancer [11, 9, 10]. Kinoshita et al. were the first to propose that LOH in the region of 9p21 was associated with progression of RCC and metastasis [9], but the study lacked long-term follow-up. Our literature search revealed several studies that reported on allelic deletion on chromosome 9p in renal cancer, but only a few studies established correlations between allelic deletion of 9p and survival with sufficient follow-up periods [11, 13]. Some of the studies relied on only one or 2 microsatellites telomeric to 9p21 to assess allelic deletion of 9p, which could have missed more centromeric LOH involving regions harbouring several tumour suppressor genes involved in cell cycle regulation. Allelic deletion of 9p was assessed by 5 markers covering the 9p21 region, as the region of interest that harbours CDKN2A/B genes, and to validate I-FISH results, which relied on a dual-colour probe with a locus-specific identifier on CDKN2A.

The follow-up period in the previous studies ranged between 31 and 48 months (median), compared to 91 months median follow-up in this study. The extended follow-up period for this cohort allowed assessment of the impact of LOH on 9p21, and especially the coding region of CDKN2A, on survival. The prognostic impact of LOH on 9p was determined by integrating it within multivariate models including the most significant variables and in externally validated prognostic models for cancer-specific survival and metastasis-free survival.

Microsatellite analysis has the advantage of detecting loss of heterozygosity in the absence of copy number variation (deletion), which is also known as copy number neutral LOH (CNNLOH). Studies on several cancers have shown that CNNLOH is a common phenomenon, but its correlation with gene expression remains poorly understood. It could still be considered to represent a mechanism of gene inactivation, especially if the wild type allele is lost and the non-functioning one is duplicated to maintain a copy neutral status [14, 15].

The incidence of CNNLOH has been studied in detail in oesophageal cancer, and it appeared to play a role in carcinogenesis and was associated with a change in gene expression levels, either by up-regulation or down- regulation. However, the authors concluded that the mechanism remains poorly understood [14, 15]. The authors suggested that CNNLOH was more common than LOH with copy number alteration in oesophageal cancer and represented 70% of all allelic deletions detected by single nucleotide polymorphism (SNP) arrays. Saeki et al. reported copy neutral LOH of the p53 locus in p53 mutant oesophageal cancer and suggested that this could be a major mechanism for inactivation of the intact allele in oesophageal squamous cell carcinogenesis associated with a mutation at p53. The authors suggested five scenarios that could result in LOH on p53; three of them are copy neutral [15].

The present study revealed CNNLOH in 9 tumours. As expected, these were not detected by I-FISH alone and hence alternate/complementary techniques are required to map these areas. On the contrary, copy number loss on chromosome 9p was detected by I-FISH alone but not by microsatellite analysis. This discordance can be explained by a number of factors; for example, contamination of tumour samples during macro-dissection (viable tumour areas were marked by a specialist pathologist on tumour blocks in this study); intratumour heterogeneity causing contamination of DNA predominantly from a sub clone with no copy number loss; or a high percentage of homozygous loss or DNA sample contamination with normal renal tissue. The lack of sensitivity of microsatellite analysis in reference to FISH to detect copy number loss LOH comes down predominantly to the superiority of FISH to cut through tumour complexity at a cellular level, allowing the observer to score regions of tumour in more than one core that has a high concentration of cells with copy number loss. This advantage is clearly lost in microsatellite analysis as it relies on DNA extracted from a macro-dissected tumour region, resulting in dilution of abnormality by a mixture of normal cells and tumour cells without copy number loss of chromosome 9p. Previous work has shown that if the proportion of tumour cells in a sample diminishes to 50% or less, the ability to detect the LOH is reduced [23]. Moreover, the discordance could have been the result of sampling bias; I-FISH results were based on the scoring of 6 different cores in contrast to three 8–10 μm curls used for DNA extraction for microsatellite analysis. The increase in the number of sampled tumour regions clearly improves the detection and identification of more subclones [24]. This could have been potentially prevented by laser microdissection of tumour tissue before DNA extraction to avoid contamination with normal renal tissue or necrotic tumour tissue, which is a potential limitation in our study.

The addition of copy number status to allelic deletion measured by LOH analysis at the CDKN2A coding region on chromosome 9p significantly enhances the predictive accuracy of the SSIGN score and Leibovich score for cancer-specific death and development of metastasis in patients undergoing resection for clinically localised renal cell carcinoma. The assessment of copy number loss with I-FISH and LOH using microsatellites is complementary for the detection of copy number aberrations and provides better prognostication data. However, discordance still exists in a number of cases for reasons explained in this study.

In conclusion, combined cytogenetic techniques improve the prognostic significance of known factors in clinically localised renal cancer following surgery. Copy number neutral LOH, seen in 10% of the cohort, carries a worse prognosis and perhaps could be a potential candidate for closer follow-up or adjuvant therapy.

## MATERIALS AND METHODS

### Study cohort

The database of Tayside Urological Cancer Network (TUCAN) databases were searched for consecutive patients who underwent radical nephrectomy for Renal Cell Carcinoma (RCC) between Jan. 2001 and Dec. 2005. This allowed each participants of the study to have atleast 10 years of follow-up. This is same cohort described previously in detail for Fluorescent *in situ* hybridization and microsatellite alteration studies [8, 16]. Ethical approval through local research committee (Ref. 12/ES/0083) was obtained prior to initiation of the study. Patients were followed up using a standard protocol based on tumour stage and grade. All tumour characteristics, clinical and follow-up data were collated for each case. Tumours were examined and re-classified according to the 2009 TNM staging [17–19]. All cases were assigned a Mayo-Clinic SSIGN score [17] and only cases with localized ccRCC were assigned a Leibovich score [20].

Follow-up was calculated from the time of surgery to the last date of assessment or date of death. The cause of death was determined based on death certificates and correspondence between clinicians and patients' general practitioners. Death from renal cancer was defined as disease-related mortality. Recurrence was diagnosed if RCC metastasis or renal bed recurrence was detected on cross-sectional imaging and confirmed in a multi-disciplinary team meeting record.

### Tissue microarray (TMA)

This was described in previous publication from the same institution [7, 19]. In summary, TMAs were constructed using Beecher^®^ arraying instrument (Beecher Instruments Inc., Sun Prairie, WI, USA) with the help of TMA Designer^®^ 2 software. Tissue cores with a diameter of 0.6 mm were punched from the marked tumour regions on paraffin blocks after being marked by the pathologist. Cores were then deposited into a master paraffin block and placed 1.2 mm apart from the neighbouring core on the x and y-axes. Sections from the resulting master paraffin block measuring 4 μm in thickness were then transferred onto glass slides to form a TMA. Each case was represented at least with six tumour cores. A TMA from normal renal tissue from the same cases was constructed to be used as a control to determine the cut-off threshold for deletion.

### Fluorescence *in-situ* hybridization (FISH)

Interphase I-FISH analysis was performed using the Vysis Locus-Specific Identifier (LSI) CDKN2A spectrum red (R)/(CEP 9) spectrum green (G) probes (Abbott Molecular, USA). The process of applying the probe to TMA slides is described in detail in our previous publication. Interpretation of I-FISH, using pre-set criteria, was carried out by 2 independent observers blinded to clinical outcomes to determine 9p status in each tumour based on 6 representative cores on the TMA and relying on control normal tissue to set threshold for deletion. Any dis-agreement was settled by reviewing slides and consensus. An algorithm describing the interpretation of I-FISH for 9p deletion was published previously [8].

### DNA extraction from formalin-fixed paraffin-embedded tissue samples

Three curls were cut from the same FFPE tumour blocks that were represented by six cores on the tumour TMAs. This was done to minimise the risk of sampling bias and reduce the effect of intratumour heterogeneity on the results.

DNA extraction was undertaken from FFPE tumour curls and corresponding normal tissue curls from the same patient using the automated EZ1 BioRobot method. This extraction protocol relies on the use of magnetic particles that bind to the DNA and are subsequently removed from the surrounding tissue by a separate magnetic source. As with the manual methods, 180 μl ATL tissue lysis buffer was added to each sample (2 × 10 μM FFPE sections) together with 20 μl Proteinase K solution. The samples were placed in an Eppendorf Thermomixer and incubated at 56°C with vigorous shaking for one hour. At the end of this period, the thermomixer temperature was increased to 90°C and incubated another hour, again with vigorous shaking. The samples were then loaded into a cartridge and placed in the EZ1 Robot. All the reagents required for the extraction of DNA from a sample are present in a single cartridge. An elution volume of 50 μl ATE buffer was used before DNA sample was stored at −20°C until use. All DNA samples concentration and quality were assessed by NanoDrop 1000 spectrometer (Thermo Fisher Scientific, Wilmington, Del).

### Microsatellites markers

Five fluorescent end-labelled microsatellite markers that map to the 9p13–21 region were used after being checked for possible single nucleotide polymorphisms (SNP). Four markers map to 9p21; D9S916, D9S1814, D9S974 and D9S942. The latter two are within the coding sequence of CDKN2A. D9S916 is telomeric to CDKN2A and CDKN2B, whereas D9S1814 is centromeric to CDKN2B. D9S171 is at 9p13 (proximal toCDKN2A/B). The details of these microsatellite markers have been described in our recent study [16].

PCR amplifications were performed in a final volume of 24ul and consisted of 10X PCR reaction buffer, 2 mM dNTPs, forward and reverse primers, HotStarTaq DNA Polymerase^®^ (Qiagen^®^) and distilled H2O. All PCRs reagents were mixed in one tube, 24 μl of the reaction mix was aliquoted into each tube pre-labelled with the sample number. The sample DNA was added (usually 1 μl; 50–200 ng) to each corresponding labelled tube and tubes placed in the thermal cycler.

The PCR programme employed consisted of 1 cycle at 95°C for 15 minutes for activation of HotStarTaq. This was followed by 35 cycles of denaturation step (94^°^C for 40 seconds), then annealing step (55°C for 40 seconds), followed by elongation or extension step (72°C for 40 second). Finally the programme terminates with 1 cycle of cooling (8°C for 7 minutes, then 4°C for 1 minute)

Following amplification, 0.5 μl of the product was denatured in deionized formamide and the fluorescent markers analysed by capillary electrophoresis. Determination of loss of heterozygosity (LOH) status was carried out by staff in the NHS Genetic Laboratory, using GeneMarker^®^ Software V2.4.0 (SoftGenetic^®^, State College, PA 16803, USA). For informative cases, the allelic loss was scored if the signal intensity of one allele was reduced by more than 40% in the tumour DNA compared to the corresponding allele in normal DNA.

### Statistical analysis

The clinicopathological data were compared based on 9p status. Proportions between categorical variables were compared using Fisher's exact and Pearson chi-square tests, as appropriate. The survival time was summarised using mean and ranges. Other continuous variables were summarised as means and standard deviation (SD) and compared using Student-*t* tests or Mann-Whitney *U* test as appropriate.

The Kaplan-Meier method was used to estimate RFS and DSS based on 9p status and other variables. The log-rank test was used to compare the survival differences between the groups.

A univariate Cox proportional hazard model was used to assess the correlation between prognostic variables and recurrence, and RCC-specific mortality. Multivariate analysis was performed for DSS and RFS after excluding the insignificant variables in univariate analysis. Backwards selection manner with the likelihood ratio criterion (for entry and removal: *p* ≤ 0.05 and *p* > 0.1 respectively) and rank of elimination was used to identify the most significant variables to be entered in the final models for RFS and DSS. The predictive accuracy of prognostic models was assessed by employing Concordance index (C-index). Statistical analysis was performed using IBM^®^ SPSS^®^ – version 21, with all tests being two-tailed and *p* < 0.05 considered statistically significant.
